# Debriefing and Learning Strategies: A Comparison between Two Reflective Analysis Styles with/without a Graphical Record of Strengths/Weaknesses

**DOI:** 10.3390/healthcare9020130

**Published:** 2021-01-28

**Authors:** Guillermo Escribano Sánchez, María Ruzafa-Martínez, César Leal-Costa, José Luis Díaz-Agea, Antonio Jesús Ramos-Morcillo, Alfonso García Sánchez

**Affiliations:** 1 Faculty of Nursing, Universidad Católica de Murcia, 30107 Murcia, Spain; gescribano@ucam.edu (G.E.S.); agarcia@ucam.edu (A.G.S.); 2 Faculty of Nursing, Universidad de Murcia, 30100 Murcia, Spain; ajramos@um.es

**Keywords:** debriefing, clinical simulation, learning strategies, motivation, nursing

## Abstract

Background: Clinical simulation efficiently complements the training of Nursing Degree students. The debriefing phase is the most important feature of simulation-based learning, where the students are able to acquire the necessary competences. It is at this stage where learning strategies and motivation play a crucial role. The objective of the study was to analyze the relationship between the style of debriefing utilized in the simulation sessions, and the learning strategies of Nursing Degree students who participated in a high-fidelity clinical simulation. Method: This was a quasi-experimental study conducted with a sample of 200 students in their third and fourth years at university. To obtain the data, an evaluation Questionnaire for the Evaluation of Learning Strategies of University Students (CEVEAPEU) was utilized, as well as two different types of structured debriefing styles, namely, with or without a graphical representation of the strengths/weaknesses during the analytical phase. The data analysis was performed with the SPSS^®^ v25 program. Results: Statistically significant differences were found, with higher scores obtained when utilizing debriefing with a graphical representation, on both scales of the questionnaire (affective and cognitive), on the motivational, metacognitive and processing, and use of information subscales, and twelve learning strategies mostly belonging to the subscales of motivation; searching, collecting, and selecting information; and processing and using information. Conclusion: Debriefing with a graphical representation is deemed, a priori, as the most adequate approach for our context, based on the greater number of learning strategies utilized by our students. The use of a written graphical record of the strengths and weaknesses in the analytical phase is recommended.

## 1. Introduction

The European Higher Education Area (EHEA) has shaped a new educational scenario in higher education [1], and the University in Spain therefore faced one of the most significant changes in its history with the Bologna Process, which signified a radical change in the structure of teaching and learning [2,3].

Aside from the theoretical framework of the knowledge harbored by the nursing discipline, it has always included a practical component, which is considered the cornerstone of its teaching. The learning of technical skills had to be adapted to this new educational context with the creation of new curricula, comprised of the different study plans used at the time. This is how the Practicum course came to be, which gathered all the practice sessions and brought together all the different methodologies and work strategies, which in essence, were the backbone of nurse training [4,5].

Motivation and learning strategies are cognitive and behavioral elements that play a fundamental role in the student’s learning [6]. Learning strategies have a close relationship with the student’s resources and capabilities, as it is the students themselves who exhaustively select them as they use them, just as with tactics, abilities, and/or learning skills. The use of the classification system developed by González and Tourón [7] was decided upon, as it was considered the most adequate based on the instrument utilized in the present study, the Questionnaire for the Evaluation of Learning Strategies of University Students (CEVEAPEU), as well as the objectives set forth. According to this classification, learning strategies are classified into three well-defined groups: cognitive strategies, metacognitive strategies, and resource management strategies.

Cognitive strategies are utilized by students to extract what is important from the information received before connecting it with the knowledge they already possess. Three types exist: review, creation, and organization. At this point, the concept of critical thinking should be highlighted, which refers to the effort made by the student to process the information obtained more profoundly and with greater reflection [8,9].

Metacognitive strategies control and regulate mental processes to achieve specific learning objectives. On the one hand, they help with choosing the time, manner, and reason for the use of specific strategies. On the other, they direct the changes needed based on the greater or lesser efficacies of the selected activities [7,8,9,10].

Resource management strategies lead to the creation of awareness in the student by learning through effects, motivation, and attitudes [6]. Within these types of strategies, we find manners in which to organize learning time and to use study environments, which should be peaceful, organized, and without distractions of any kind. For effective learning, content and strategies are needed, with both being equally important. Strategies secure knowledge in the long term, a process that is vitally important due to its interaction with existing knowledge [9,10].

The motivations, as well as the intentions and objectives, of the students are essential elements in any type of strategic behavior, which determine what specific strategies are utilized in each type of specific learning. As related to learning, motivation can be defined as the action of providing reasons stimulating the will to learn. There are two types of motivation: intrinsic and extrinsic. Intrinsic motivation considers each specific activity an end in itself, meaning that it is not used to achieve a different objective. The student who has this type of motivation has an attitude that propitiates a great mental effort and, as a result, achieves a deeper and more effective type of learning. Extrinsic motivation, on the other hand, considers each task as a fundamental element for achieving other objectives. The student performs the activities for reasons that are not related to the task itself but for achieving the objectives proposed [11,12].

In essence, the motivated student strives until the end, and the one who is not cannot bear the intrinsic effort throughout the entire learning process. Therefore, the students themselves play a fundamental role in the training of a new Nursing professional, as the ones responsible for their own learning. The Practicum then becomes the axis around which the curriculum of the Degree is structured, where knowledge is integrated and transferred through clinical practice [13,14].

In this new scenario, clinical practice is becoming more reflective and examining the actions of the professionals themselves. When thinking about a specific task, a practical investigation is conducted, which helps us to solve real-life situations, meaning that at the same time that an activity is being performed, one must understand what occurred and, if necessary, change what was done incorrectly [15].

The learning process always starts from a specific experience that the student examines through reflection, which allows a process to start for creating and formulating a series of concepts that will become useful in later situations experienced, initiating new experiences and returning to the initial step. Therefore, so that reflective learning is produced, two fundamental conditions are needed, which are found in any learning process: the perception and processing of information. Both are shaped by perceptions through a real experience, which is based on emotions, or through an abstract experience, shaped by thoughts, in a continuous process of active experimentation or observation through reflection [16,17].

Learning, in essence, is the end result of the manner of feeling and creating the information obtained of each student, as the perception and the processing of information are two different styles of learning, which undoubtedly determines the learning method of each particular student [18].

As previously mentioned, the current practical training is conducted through different clinical practices, through which the Nursing curriculum is entirely structured. This brings together theory and practice into a close-knit relationship, with a reflective process found in each action performed, thereby shaping the practical knowledge, which would be impossible to acquire otherwise [5].

Within the Practicum, which is completely integrated into the curriculum, the clinical simulation has been covering the training needs of the Nursing Degree students. This teaching methodology guarantees that the students acquire the necessary knowledge and skills in a scenario that is safe for them, achieving very high levels of satisfaction according to the students. Additionally, it is very useful for the development of communication skills with the multidisciplinary team, increasing confidence and effectiveness in any health organization, and ultimately achieving a nexus between theoretical knowledge and real-world clinical practice [19,20,21].

A clinical simulation is composed of very defined phases: prebriefing, or a session beforehand (where the simulation guidelines are established, a climate of trust is created, and a fictional contract is agreed upon between the participants and the facilitator); the simulation part of the clinical simulation; and the debriefing, where a reflection is made about what has occurred, and where the real learning takes place [22].

In the debriefing phase, a reflection and an analysis of the simulated clinical scenario are performed, to analyze the performance and to maintain or improve the future actions of the students. The debriefing phase is considered the most important stage within the simulation for achieving effective and significant learning. The importance of effective learning is fundamental when establishing a type of learning that is based on experience through clinical simulation [23].

Debriefing must be a self-evaluation process, meaning that the students themselves, helped by a facilitator, must point out their strengths and weaknesses with respect to their behavior during the simulation. In this way, what was performed correctly is reinforced, and an inquiry is made about the causes behind inadequate or erroneous actions. In the health sciences, the debriefing stage is conducted in a structured manner and is usually composed of three phases: description, analysis, and application. In the description phase, the participants describe what was experienced and what they felt during the scenario. The analysis phase is focused on the strengths and weaknesses of the participants, and the application phase helps the students to bring what was learned in the simulated clinical scenario to real life [24,25,26].

The clinical simulation facilitator is in charge of obtaining a high degree of motivation of the students, making them the leading actors in their own learning through the experiences lived, the search for information, critical analysis, reflection, and the integration of all the knowledge obtained. The role of the facilitator demands that he or she is a well-trained educator who can promote self-evaluation among and feedback from the students. Therefore, his or her actions will greatly help with the acquisition of competencies, and are key elements for the debate and the contrasting of ideas during the debriefing session [27,28,29].

Thus, it is in the debriefing phase when the students can be greatly motivated, and as a result, the facilitator can help the students to utilize their learning strategies. This will promote learning and ultimately achieving a type of learning that is of higher quality and longer lasting through time.

Structured debriefing with the support of visual elements (usually video analysis) positively helps with the learning results, acquisition of competencies, problem resolution skills, assimilation of knowledge, and clinical thinking and reasoning. The introduction of visual elements helps to increase the quality of the debriefing, as well as the confidence, satisfaction, and skills of the students [30,31].

It has been observed that debriefing with audiovisual support results in an improvement in the development of clinical and psychomotor skills, coupled with a high efficacy when obtaining the student’s satisfaction, greater and better critical thinking skills, and an increase in the clinical thinking and knowledge of the student, when compared with debriefing that utilizes only a verbalized discussion [31,32,33,34].

In our experience with simulation within the Nursing Degree at the UCAM, we have found that a few facilitators in the analytical phase, when the successes and the errors of the students are presented, utilized the blackboard so that the students who had been part of the simulated case could write down their strengths (pluses) and weaknesses (deltas). Afterwards, the facilitators work with the students to reinforce the strengths and reflect on the causes behind the weaknesses, and discuss how these weaknesses should change in future cases. Other instructors, however, do not use the blackboard to work on the strengths and weaknesses, but instead only verbalize them and work with the students only in a spoken manner. The hypothesis of the present work is that the students who reflect on their behaviors, helped with the graphical representation, take greater advantage of the session, as shown by an improvement in the learning strategies utilized.

The objective of the study was to analyze the existing relationship between the type of structured debriefing utilized (with or without a graphical representation of the strengths and weaknesses) and the learning strategies utilized by the third- and fourth-year students enrolled in the Nursing Degree at the Catholic University of Murcia (UCAM).

## 2. Materials and Methods

### 2.1. Design

A quasi-experimental study was conducted with quantitative research techniques, with a final measurement conducted at the end of the simulation sessions (posttest).

### 2.2. Sample

The assignment to the different debriefing types was performed based on the order of the group sessions taught by the Nursing Practices Unit from the University when conducting clinical simulations. The debriefing without a graphical representation (DWOGR) was used with the odd-numbered student groups, and the debriefing with a graphical representation (DWGR), with the even-numbered groups.

The inclusion criterion was all the students who took part in clinical simulation sessions during the period studied.

The exclusion criteria were voluntary refusal to participate in the study and not completing all the clinical simulation sessions.

The starting sample size was 204 students; however, 4 students did not complete the simulation sessions and were excluded from the study. Thus, the final sample size was 200 students.

### 2.3. Instruments

The instrument utilized was the Questionnaire for the Evaluation of Learning Strategies of University Students (CEVEAPEU), validated with Spanish university students [35].

This questionnaire is a self-report type of questionnaire and is composed of 88 items divided into two scales, six subscales, and twenty-five learning strategies (Table 1).

The first scale encompasses affective, support, and control strategies. It includes four subscales: motivational strategies; affective strategies; metacognitive strategies; and strategies for the control of the context, social interactions, and management of resources. It includes sixteen strategies, which initiate and maintain the learning process.

The second scale contains the cognitive strategies. It is divided into two subscales: search strategies and strategies for the acquisition and selection of information, and strategies for processing and using the information. It comprises nine strategies, which are responsible for the global processing of information.

The responses to the items were scored with a Likert-type scale with 5 response options, whose values could be complete disagreement (1), disagreement (2), undecided (3), in agreement (4), and in complete agreement (5). Some questions raised in the items of the questionnaires were, for example, “when I study I do so due to my interest in learning”, “my academic performance is dependent on my effort”, “I know what the objectives of the courses are”, etc.

This questionnaire was utilized, as it was created and validated with Spanish university students. Additionally, the questionnaire used added a subscale related to the search for, acquisition, and selection of information. It clearly included all the components of planning, knowledge about the objectives, evaluation criteria, self-evaluation, and control or self-regulation within the metacognitive strategies. It also included more components within the concept of intelligence as being modifiable, and the attributions and motivation strategies. It had a strong internal consistency, as evidenced by the reliability analysis with the use of Cronbach’s alpha, with a value of 0.897 for the questionnaire as a whole. On the affective scale, a result of 0.819 was obtained, and on the cognitive scale, a value of 0.864. For the subscales, the obtained results ranged from 0.692, for the motivation strategies, to 0.821, for the processing and use of information strategies. The results for the different strategies ranged between 0.500, corresponding to an intrinsic motivation strategy, and 0.771, corresponding to the strategy of the organization of information [35].

### 2.4. Procedure

The study was conducted in the Clinical Simulation classroom at the UCAM during the academic term, between October, 2016, and July, 2017, by two instructors. One of them conducted the simulation sessions with the fourth-year students, and another one, with the third-year students.

The instructors involved in the study met on various occasions before the start of the study to unify their criteria when performing the clinical simulation sessions and the debriefing.

The students completed all the clinical simulation sessions planned for each academic year, divided into groups of 12–15 students.

The clinical simulation sessions were planned equally for all the groups from the same academic year: prebriefing, clinical simulation (simulated clinical scenarios), and debriefing.

The debriefing session conducted was structured into three phases: description, analysis, and application. The description and application phases of the debriefing were conducted identically for all the student groups. However, both instructors conducted the debriefing without a graphical representation in the analysis phase with the odd-numbered groups, and the debriefing with a graphical representation with the even-numbered groups.

The different debriefing conditions utilized are described below:

Condition 1: in the analytical phase of the debriefing without graphical representation, the instructors fomented debate, discussion, and reflective analysis among all the students, verbalizing the strengths and weaknesses detected in the simulated clinical scenario (plus/delta), but without writing anything on the board.

**Figure 1:**
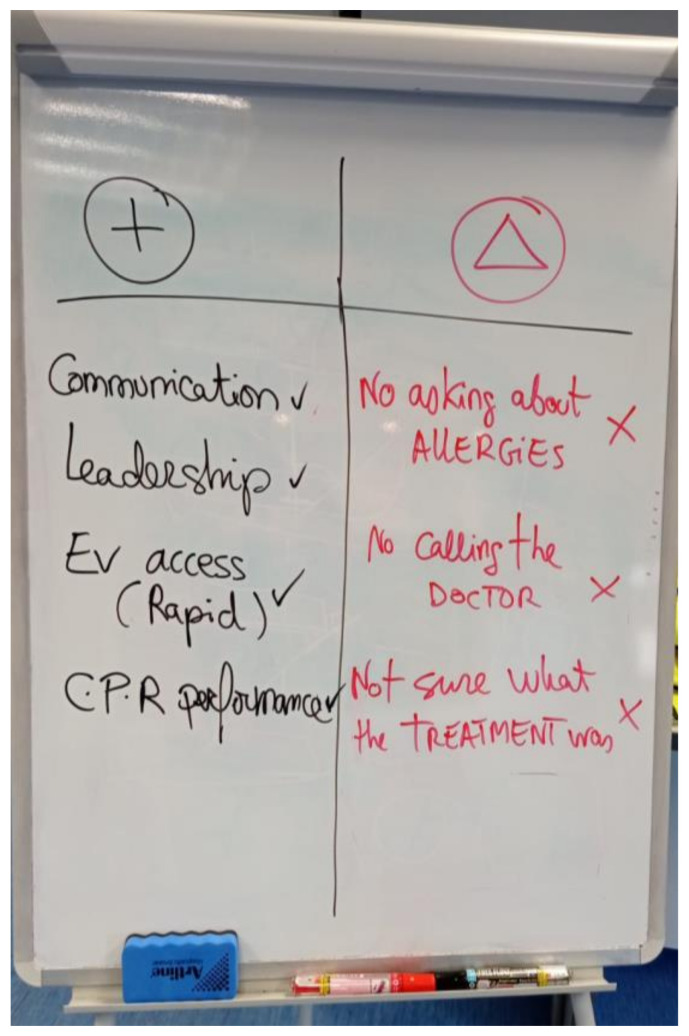
Example of graphical representation of strengths/weaknesses (plus/delta).

Condition 2: in the analytical phase of the debriefing with graphical representation, the instructors also fomented reflective analysis, discussion, and open debate, verbalizing the strengths and weaknesses found during the simulated clinical scenario (plus/delta), and at the same time, the students who took part in the simulation wrote these on the board (Figure 1).

The data were collected by the instructors involved under strict supervision, after the last session of the simulation training program, which consisted of a weekly 4 h session over several months. The students were asked to indicate the learning strategies they had utilized during the clinical simulation sessions. The questionnaire was provided as paper copies to the participants to complete, assigning random numbers to them to ensure the confidentiality of the data at all times.

### 2.5. Statistical Analysis

To process the information, a database was created with the SPSS^®^ v25 program (SPSS Inc., Chicago, IL, USA). Afterwards, a statistical analysis was performed with different statistical tests.

The mean, standard deviation, asymmetry, and kurtosis were calculated for all the scales, subscales, strategies, and items from the Questionnaire for the Evaluation of Learning Strategies of University Students (CEVEAPEU).

The existing relationships were analyzed between the type of debriefing utilized and the scales, subscales, and strategies from the CEVEAPEU questionnaire. Student’s t test was utilized for independent samples, and for dichotomous quantitative and qualitative variables.

Before the Student’s t test was performed, the assumption of the normality of the quantitative variables was verified with the Kolmogorov–Smirnov test, which measured the agreement between the distribution of the sample and a normal distribution. Additionally, the homogeneity of the variances of the variables to be compared was verified with Levene’s test.

### 2.6. Ethical Considerations

The participants signed an informed consent form, thus providing their authorization for the use of the data collected for research purposes.

The study was evaluated and approved by the Ethics Committee from the Catholic University of Murcia (UCAM) (reference number: 5939).

## 3. Results

The total sample comprised 21.5% men and 78.5% women. Of these, 40.5% were third-year and 59.5% were fourth-year Nursing Degree students. Additionally, 48% took part in the debriefing without a graphical representation of the strengths/weaknesses in the analytical phase (only verbalized) (DWOGR), and 52% took part in the debriefing with a graphical representation in the analytical phase (DWGR).

### 3.1. Test Results

The average score of the students obtained for the affective, support, and control strategies was 56.102, and that for the cognitive strategies was 29.673, with both scales showing asymmetry and kurtosis values very close to zero (0 ± 0.4), which indicates a normal distribution (Table 2).

### 3.2. Relationship between Motivation and Learning Strategies According to the CEVEAPEU and the Type of Debriefing Utilized

After analyzing the differences between the means for the motivation and the learning strategies of the students, according to the type of debriefing (DWOGR or DWGR), the following results were observed:

On the affective scale, a higher score was observed when using DWGR, with a difference in means of −0.533. On the cognitive scale, a higher score was also found when using DWGR, with a difference in means of −0.852 (Table 3).

For the motivation subscale, the highest score was found with DWGR, with a difference in means of −0.273. Likewise, the metacognitive subscale, and the subscale of the processing and use of information, showed higher scores when using DWGR, with differences in means of −0.248 and −0.267, respectively. These differences were statistically significant (Table 3).

As for the strategies utilized, higher scores were observed with the DWOGR for the following ones: skills, social interaction and learning with peers, and the control of the context. On the other hand, higher scores were found with DWGR for the following strategies: self-efficacy and expectations, the value of the task, internal attribution, knowledge of the objectives and evaluation criteria, self-evaluation, intrinsic motivation, the organization of information, personalization and creativity, critical thinking, the acquisition of information, the elaboration of information, storage, memorization, the use of mnemonic rules, and the use and transfer of information acquired. These differences were statistically significant (Table 3).

## 4. Discussion

After the analysis, statistically significant differences were observed for the variable DWGR/DWOGR on both scales of the questionnaire (affective and cognitive), on the motivational, metacognitive and processing, and use of information subscales, and twelve learning strategies mostly belonging to the subscales of motivation; searching for, collecting, and selecting information; and processing and using information. Although the benefits obtained from the use of a structured debriefing after a simulation experience have already been greatly discussed [30,31,32], this study also reveals that the graphical representation during the analytical phase of the debriefing is related to a greater use of learning strategies by the students. This could encourage the facilitators to not only use their voice in the analytical phase of the debriefing, as it could be complemented with a graphical representation of the positive aspects of the students in the simulation experience, as well as those that should be improved.

Despite performing an exhaustive bibliographic search, no studies were found that compared any type of debriefing with the learning strategies of students, so the comparison of our results with similar studies was not possible.

In the present study, two types of structured debriefing were utilized, which are commonly used in clinical simulations in the health sciences. In the literature consulted, an important influence was found for the use of structured debriefing, with a substantial improvement of the critical thinking and clinical reasoning of the students, as well as the achievement of significant learning and a greater motivation to obtain skills related to the resolution of problems and the making of more adequate clinical decisions [30].

Through the use of the questionnaire for learning strategies ACRA-A (a questionnaire for strategies of the acquisition, coding, recovery, and support of information), the research by Bertel and Martinez with students from Bolivia, and the research by Garzuzi and Mafauad with students in Argentina, showed, on the one hand, results indicating a more frequent use of support strategies as opposed to cognitive ones [36,37]. On the other hand, in the present study, the students utilized support and cognitive strategies to the same degree, without the predominance of any of the scales.

The study by Rinaudo, Chiercher, and Donolo evidenced a greater use of strategies of organization, creation, and the regulation of effort, which are encompassed within the metacognitive strategies. Those that were used to a lesser degree were the strategies of reviewing and critical thinking, included in the cognitive strategies [38]. However, the results of the present study show that the students utilized the cognitive learning strategies to a greater extent when compared to the metacognitive ones.

For Gargallo, the learning strategies that were most utilized by the students in the Spanish context were self-efficacy/expectations, intrinsic motivation, the value of the task, extrinsic motivation, physical/mood state, anxiety, planning, control/self-regulation, knowledge about the objectives and evaluation criteria, social interaction skills, the control of the context, the selection of information, knowledge of sources and searching for information, the acquisition of information, the transfer/use of information, and storage/simple repetition [39]. However, in the present study, the students did not use the following learning strategies: extrinsic motivation, physical and mood state, anxiety, planning, control and self-regulation, knowledge of sources and searching for information, the selection of information, and storage through simple repetition.

Real-world clinical practices play an important role in the use of the learning strategy of the control of the context in the simulation sessions, due to the knowledge already possessed and used by the students in their day-to-day work in health centers, as compared to those students who had not experienced this type of real-world practice [38,40,41]. The present study also showed a similar result, but with differences in means that were higher when using DWGR with respect to DWOGR. This is paradoxical, as all the third- and fourth-year students enrolled in the Nursing Degree had previously taken part in clinical practice.

Lastly, the research by Garcia showed that the learning strategies that were most utilized by the students at the end of the clinical simulation sessions were similar to those from our study. Thus, clinical simulations have been shaped as an efficient tool for the training of new health professionals, through the improvement of the learning strategies of the participants [42].

In this research study, the results show that the students did not utilize the following strategies: the management of resources, external attribution, control/self-regulation, knowledge of the sources and searching for information, and the selection of information. On the other hand, they utilized the following ones: the control of the context, internal attribution, knowledge of the objectives and evaluation criteria, the acquisition of information, the elaboration of information, storage, memorization, the use of mnemonic rules, and the use and transfer of acquired information.

After the analysis of the data obtained, it was observed that most of the learning strategies with statistically significant differences with respect to the type of debriefing utilized showed higher mean values for DWGR. We believe that the use of the plus/delta tool through a graphical representation in the analytical phase greatly contributed to a debriefing that was more systematized within its structure. This helped the students to find strengths and weaknesses, activating and utilizing a greater quantity of learning strategies and thus achieving more profound reflection and, therefore, greater learning, as opposed to only verbalizing them. The results can be explained in accordance with the cognitive variables that had an influence on the attention paid by these students when the information had a visual (iconic) and aural (echoic) format, as compared with when this information was only presented in a spoken manner.

The visual presentation of the information greatly facilitates its learning, as the use of graphical language helps the students to become more aware of this information, facilitating its learning and greatly improving the learning methodology, which results in the optimization of the learning process [43].

Lists of words and phrases are optimally learned when the information is created by the individuals themselves as opposed to being obtained by listening to others. In this sense, it is important to underline the fundamental importance of motivation in learning. The stimulation of inquiry produces an activation of specific brain circuits, increasing the activity of the hippocampus, which is intimately involved in learning and memory [44].

Once the existing relationship between the learning strategies and the type of debriefing utilized (DWGR/DWOGR) is analyzed, future studies should perform an in-depth analysis of the reasons behind the relationship between the learning strategies described and the type of debriefing utilized (DWGR/DWOGR), with other health sciences students (medicine, physiotherapy, etc.) who take part in clinical simulations.

As possible limitations, we know that when only analyzing the debriefing variable, we do not know if there is another variable that has an influence on the use of learning strategies by the students. It would be interesting to analyze if the graphical plus/delta tool utilized in the DWGR directly affects their use, in order to optimize learning by using the most appropriate type of debriefing. Another limitation derived from the evaluation instrument utilized is the low values of Cronbach’s alpha (<0.60) for the following learning strategies: intrinsic motivation, extrinsic motivation, external attributions, internal attributions, the concept of intelligence as modifiable, self-evaluation, and the management of resources for the efficient use of information. The results found for these strategies should be interpreted with caution. Lastly, the use of two facilitators is a possible non-controlled variable, as the interaction dynamics in the debriefing session could have varied depending on the facilitator who directed the session, even though the facilitators had met on various occasions to unify the criteria utilized when conducting the clinical simulation and debriefing sessions.

## 5. Conclusions

The DWGR is defined, a priori, as the most adequate approach, based on the greater number of learning strategies utilized by the students.

We therefore recommend that the simulation facilitators use a graphical representation in the analytical phase of the debriefing session when discussing the strengths and weaknesses of the students’ behavior, in light of the results of the present study.

## Figures and Tables

**Table 1:** Structure of the Questionnaire for the Evaluation of Learning Strategies of University Students (CEVEAPEU). Reliability data [35].

Scales	Subscales	Strategies
Affective, support and control (α = 0.819)	Motivational (α = 0.692)	Intrinsic motivation (α = 0.500)
		Extrinsic motivation (α = 0.540)
		Value of the task (α = 0.692)
		External attributions (α = 0.539)
		Internal attributions (α = 0.537)
		Self-efficacy and expectations (α = 0.743)
		Concept of intelligence as modifiable (α = 0.595)
	Affective (α = 0.707)	Physical state and state of mood (α = 0.735)
		Anxiety (α = 0.714)
	Metacognitive (α = 0.738)	Knowledge of objectives and evaluation criteria (α = 0.606)
		Self-evaluation (α = 0.521)
		Planning (α = 0.738)
		Control and self-regulation (α = 0.660)
	Control of the context, social interaction and management of the resources (α = 0.703)	Control of the context (α = 0.751)
		Social interaction skills and learning with peers (α = 0.712)
		Management of resources for the efficient use of information (α = 0.500)
Cognitive (α = 0.864)	Search, gathering and selection of information (α = 0.705)	Knowledge of sources and search for information (α = 0.685)
		Selection of information (α = 0.630)
	Processing and use of information (α = 0.821)	Acquisition of information (α = 0.677)
		Elaboration of information (α = 0.739)
		Organization of the information (α = 0.692)
		Personalization and creativity. Critical thinking (α = 0.771)
		Storage, memorization, use of mnemonic techniques (α = 0.765)
		Storage through simple repetition (α = 0.691)
		Use and transfer of the information acquired (α = 0.656)

**Table 2:** Descriptive statistics of the Scales, Subscales, and Strategies of the CEVEAPEU.

Scales	Mean	Std. Dev.	Asymmetry	Kurtosis
Affective, support and control strategies	56.102	4.331	−0.072	−0.098
Cognitive strategies	29.673	3.059	−0.265	−0.110
**Subscales**	**Mean**	**Std. Dev.**	**Asymmetry**	**Kurtosis**
Motivational strategies	24.886	2.493	0.337	0.085
Affective strategies	4.001	1.665	−0.009	−0.384
Metacognitive strategies	15.431	1.664	0.274	0.080
Social context strategies	11.783	1.401	−0.360	0.001
Search and selection strategies	7.088	1.025	0.135	−0.263
Processing and use strategies	22.584	2.497	−0.287	0.028
**Strategies**	**Mean**	**Std. Dev.**	**Asymmetry**	**Kurtosis**
Planning	3.168	0.767	−0.121	0.164
Social interaction	3.908	0.441	−0.617	0.416
Self-efficacy and expectations	4.291	0.433	−0.011	−0.449
Anxiety	3.400	0.966	−0.273	0.768
Physical state	3.771	0.683	−0.466	−0.004
Value of the task	4.416	0.459	−0.749	0.987
Control of the context	4.063	0.495	−0.842	0.846
Control and self-regulation	4.059	0.500	−0.436	0.387
Internal attribution	4.278	0.464	−0.396	0.659
External attribution	2.667	0.898	−0.077	−0.483
Evaluation O\objectives	4.127	0.525	−0.107	−0.285
Intelligence as modifiable	4.375	0.739	−0.330	0.352
Extrinsic motivation	2.295	0.977	0.405	−0.493
Self-evaluation	4.153	0.443	0.484	−0.199
Intrinsic motivation	4.435	0.415	−0.469	−0.421
Organization of information	3.933	0.717	−0.885	0.943
Personalization and creativity	3.959	0.339	0.219	0.957
Acquisition of information	3.676	0.565	0.250	0.406
Elaboration of information	4.076	0.570	−0.389	0.490
Storage and memorization	3.941	0.733	−0.926	0.285
Search for information	3.420	0.723	−0.225	−0.249
Selection of information	3.668	0.522	−0.230	0.163
Transfer and use	4.098	0.474	0.384	0.085
Storage and repetition	2.487	1.044	0.260	−0.172
Management of resources	3.770	0.764	−0.197	0.257

Note. Std. Dev.: Standard deviation. Created by author.

**Table 3:** Differences in means, with Student’s t test for independent samples, between motivation and learning strategies according to the CEVEAPEU and the type of debriefing.

	Debriefing Dwogr		Debriefing Dwgr					
	Mean	Std. Dev. Típica	Mean	Std. Dev. Típica	t	p	Confidence Interval 95%	
Affective scale	55.848	4.534	56.381	4.534	−0.168	0.027	−1.315	−0.508
Cognitive scale	29.004	2.941	29.856	3.168	0.811	0.042	−0.502	−0.106
Motivational sub	24.750	2.365	25.023	2.617	0.076	0.035	−0.670	−0.224
Affective sub	3.874	1.781	4.118	1.551	−1.037	0.301	−0.709	0.220
Metacognitive sub	15.312	1.765	15.560	1.563	1.055	0.039	−0.215	−0.012
Social inter. Sub	11.713	1.433	11.874	1.374	−0.675	0.500	−0.525	0.257
Information search sub	7.132	0.982	7.048	1.066	0.583	0.561	−0.202	0.371
Information processing sub	22.456	2.380	22.723	2.606	0.754	0.043	−0.431	−0.264
Planning str.	3.234	0.945	3.108	0.554	1.162	0.247	−0.087	0.280
Social inter. Str.	4.153	0.346	3.682	0.398	8.886	0.019	0.366	0.575
Self-efficacy Str.	4.023	0.342	4.538	0.354	−9.429	0.020	−0.612	−0.417
Anxiety str.	3.309	0.945	3.483	0.981	−1.282	0.201	−0.444	0.094
Physical state str.	3.700	0.646	3.836	0.712	−1.410	0.160	−0.326	0.054
Value of Task Str.	4.194	0.494	4.620	0.307	−7.370	0.031	−0.539	−0.311
Control context Str.	4.320	0.467	3.778	0.357	−9.366	0.002	−0.663	−0.462
Control and self-regulation Str.	4.069	0.505	4.049	0.498	0.275	0.783	−0.120	0.159
Internal attrib. Str.	4.079	0.435	4.462	0.412	−6.389	0.022	−0.501	−0.265
External attrib. Str.	4.023	0.342	4.538	0.354	−0.248	0.804	−0.283	0.219
Knowledge str.	3.755	0.390	4.471	0.380	9.125	0.005	−0.823	−0.608
Mod. Intelligence str.	4.406	0.689	4.346	0.785	0.573	0.567	−0.166	0.266
Extrinsic motivation str.	2.339	0.993	2.254	0.965	0.607	0.544	−0.189	0.357
Self-evaluation Str.	3.851	0.245	4.432	0.399	−8.267	0.025	−0.674	−0.487
Intrinsic motivation str.	4.248	0.409	4.606	0.342	−6.729	0.008	−0.463	−0.253
Information org. Str.	3.689	0.732	4.157	0.626	−4.866	0.003	−0.657	−0.278
Personalization str.	3.768	0.272	4.134	0.299	−9.009	0.026	−0.445	−0.285
Information acquisition str.	3.284	0.410	4.039	0.430	−7.651	0.012	−0.872	−0.636
Elaboration of Information Str.	3.846	0.542	4.288	0.512	−5.936	0.021	−0.589	−0.294
Storage memorization str.	3.659	0.750	4.202	0.615	−5.607	0.007	−0.733	−0.351
Search for Information Str.	3.442	0.665	3.399	0.775	0.426	0.671	−0.158	0.245
Selection of information Str.	3.690	0.520	3.649	0.525	0.555	0.580	−0.104	0.187
Transfer and use Information Str.	3.792	0.295	4.381	0.432	−9.164	0.015	−0.693	−0.485
Storage repetition str.	2.375	1.005	2.591	1.074	−1.467	0.144	−0.507	0.074
Resource management str.	3.734	0.746	3.802	0.783	−0.632	0.528	−0.282	0.145

Note. Std. Dev.: Standard deviation. DWOGR: Debriefing without a graphical representation. DWGR: debriefing with a graphical representation. Created by author.

## Data Availability

The data presented in this study are available on request from the corresponding author. The data are not publicly available due there are unpublished results.
